# FRAILTY AND SOCIOECONOMIC STATUS IN BRAZIL, CHINA, AND INDIA

**DOI:** 10.1093/geroni/igac059.1499

**Published:** 2022-12-20

**Authors:** Benjamin Seligman, Arunika Agarwal, David Bloom

**Affiliations:** David Geffen School of Medicine at UCLA, Los Angeles, California, United States; Department of Global Health and Population, Harvard T.H. Chan School of Public Health, Boston, Massachusetts, United States; Department of Global Health and Population, Harvard T.H. Chan School of Public Health, Boston, Massachusetts, United States

## Abstract

Frailty, the vulnerability to poor health outcomes following physiologic stress, acts as a measure of physiological age that can be used to compare the health of older populations. Using a frailty index validated in multiple countries, we looked at the relationship between four measures of socio-economic status (SES) and frailty in Brazil, China, and India. For SES measures, we considered within-country income and wealth deciles, education level, and rural residence. Analysis used beta regression, first within each country, then with combined data with country fixed effects and country-by-SES interactions. We found evidence of strong, statistically significant negative associations between education level and frailty in all three countries. Associations between frailty and income or wealth decile or rural residence were small and inconsistently statistically significant. This demonstrates the key role of education for healthy aging and the value of harmonized frailty measures for comparative studies of older populations.

